# Overexpressing *OsPIN2* enhances aluminium internalization by elevating vesicular trafficking in rice root apex

**DOI:** 10.1093/jxb/erv385

**Published:** 2015-08-06

**Authors:** Daoming Wu, Hong Shen, Ken Yokawa, František Baluška

**Affiliations:** ^1^College of Agriculture, South China Agricultural University, Guangzhou 510642, PR China; ^2^Department of Plant Cell Biology, IZMB, Univerisity of Bonn, Bonn D-53115, Germany

**Keywords:** Al internalization, aluminium, endocytosis, *Oryza sativa* L., *OsPIN2*, vesicle.

## Abstract

Aluminium (Al) sequestration is required for internal detoxification of Al in plant cells. In this study, it was found that the rice *OsPIN2* overexpression line (OX1) had significantly reduced Al content in its cell wall and increased Al concentration in cell sap only in rice root tips relative to the wild-type (WT). In comparison with WT, OX1 reduced morin staining of cytosolic Al, enhanced FM 4–64 staining of membrane vesicular trafficking in root tip sections (0–1mm), and showed morin-FM 4–64 fluorescence overlap. Recovery treatment showed that cell-wall-bound Al was internalized into vacuoles via endocytic vesicular trafficking after removal of external Al. In this process, OX1 showed a higher rate of Al internalization than WT. Brefeldin A (BFA) interfered with vesicular trafficking and resulted in inhibition of Al internalization. This inhibitory effect could be alleviated when BFA was washed out, and the process of alleviation was slower in the cells of WT than in those of OX1. Microscopic observations revealed that, upon Al exposure, numerous multilamellar endosomes were detected between the cell wall and plasma membrane in the cells of OX1. Moreover, more vesicles enriched with Al complexes accumulated in the cells of OX1 than in those of WT, and these vesicles transformed into larger structures in the cells of OX1. Taken together, the data indicate that endocytic vesicular trafficking might contribute to Al internalization, and that overexpressing *OsPIN2* enhances rice Al tolerance via elevated endocytic vesicular trafficking and Al internalization.

## Highlight

Overexpression of an auxin efflux carrier gene, *OsPIN2*, enhanced rice aluminium tolerance via elevated endocytic vesicular trafficking and aluminium internalization in rice root apex.

## Introduction

Aluminium (Al) toxicity is a major factor limiting crop growth and production in acid soils (Kochian *et al.*, 2015). The most dramatic symptom of Al toxicity is the inhibition of root growth, which is caused mainly by the Al-induced inhibition of cell elongation and division (Ma *et al.*, 2014). Al is so reactive that it can bind to multiple sites including the cell wall, plasma membrane, cytoskeleton, and nucleus, and then affects their functions (Panda *et al.*, 2009; Matsumoto and Yamamoto, 2013; Ma *et al.*, 2014). In order to survive in Al toxic, acidic soils, plants have developed several Al resistance mechanisms, which are categorized as external detoxification and internal tolerance (Ma *et al.*, 2014; Kochian *et al.*, 2015). The well-studied plant strategy of external detoxification is where roots can exclude Al by secreting organic acids (Ma *et al.*, 2014; Kochian *et al.*, 2015). In contrast, the internal tolerance strategies allow the plant to tolerate Al accumulation either in the root cell wall by binding Al to pectin and hemicellulose (Schmohl and Horst, 2000; Yang *et al.*, 2011; Zhu *et al.*, 2012), or in root symplasm via Al uptake and chelation/sequestration (Xia *et al.*, 2010; Huang *et al.*, 2012; Kochian *et al.*, 2015). Compared with Al accumulation in the cell wall, Al complexation and sequestration within symplasm is a more effective strategy to detoxify Al (Xia *et al.*, 2010), because Al accumulation in the cell wall can inhibit root growth by changing cell wall components and limiting its extensibility (Ma *et al.*, 2004; Yang *et al.*, 2008; Zhu *et al.*, 2012).

Among the small-grain cereal crops, rice (*Oryza sativa*) is characterized as the most Al-tolerant species due to its excellent internal tolerance strategies (Ma *et al.*, 2014; Kochian *et al.*, 2015). Over the past 5 years, two transporters that function cooperatively and are required for internal detoxification of Al in rice have been reported by Ma and co-workers. One of them is an Al^3+^-specific Nramp transporter, Nrat1, which contributes to Al^3+^ uptake across the root plasma membrane (Xia *et al.*, 2010). The other is a tonoplast-localized half-size ABC transporter, OsALS1, which mediates vacuolar sequestration of Al (Huang *et al.*, 2012). Previous results showed that *OsPIN2* overexpression lines (OXs) had significantly increased Al concentration in cell sap and reduced Al content in the cell wall in root apexes of rice relative to the wild-type (WT) (Wu *et al.*, 2014). However, the expression of *Nrat1*and *OsALS1* was not different between the WT and OX lines, indicating *Nrat1* and *OsALS1* had little contribution to the difference in Al translocation between OX and WT.

Auxin plays rather multipurpose roles in Al resistance responses, including a function as a signal molecule to respond to Al stress (Matsumoto and Yamamoto, 2013; Kochian *et al.*, 2015), and regulating Al distribution within cell (Zhu *et al.*, 2013). PIN proteins, auxin efflux facilitators, direct the polar auxin transport and the asymmetric auxin distribution (Adamowski and Friml, 2015). These proteins rapidly and reversibly cycle between the plasma membrane and endosomes via vesicle trafficking (Kleine-Vehn *et al.*, 2008; Adamowski and Friml, 2015). Among these proteins in *Arabidopsis*, PIN2 expression is the only root apex specific protein, and localizes predominantly in the epidermal and cortical cells of the root apex transition zone (Blilou *et al.*, 2005; Verbelen *et al.*, 2006), which also is the most Al-sensitive portion of root (Sivaguru *et al.*, 1999; Kopittke *et al.*, 2015). Thus, PIN2 may respond to Al stress easier and earlier than other PIN proteins (Shen *et al.*, 2008; Sun *et al.*, 2010). Shen *et al.* (2008) found that Al treatment increases PIN2 transcript levels, and modulates the recycling of PIN2-containing endocytotic vesicles between the plasma membrane and endosomes. Furthermore, PIN2 vesicle trafficking was rather frequent in the transition zone (Verbelen *et al.*, 2006; Baluška *et al.*, 2010; Baluška and Mancuso, 2013), which also accumulated more Al than other zones in *Arabidopsis* root, indicating that these PIN2 vesicles might contribute to the Al internalization (Shen *et al.*, 2008; Sun *et al.*, 2010). Therefore, an important question remains open. Is PIN2 vesicle trafficking involved in the change of Al internalization mediated by overexpressing OsPIN2?

Endocytosis is an important internalization pathway for the intracellular uptake of portions of plasma membrane and extracellular cargos via pinching off of vesicles from the plasma membrane (Šamaj *et al.*, 2004). Studies have shown that vesicular trafficking might be one of the earliest targets of Al toxicity in the root apexes (Amenós *et al.*, 2009; Krtková *et al.*, 2012); moreover, endocytosis might be involved in Al internalization (Illéš *et al.*, 2006).

In this study, the relationship between vesicle trafficking and Al internalization was investigated by using rice ‘Nipponbare’ (WT) and *OsPIN2*-overexpressing transgenic plants (OX1), and attempted to explore the underlying mechanism whereby overexpressing *OsPIN2* could affect Al distribution in the rice root apex.

## Materials and methods

### Plant materials and growth conditions

The rice ‘Nipponbare’ (*Oryza sativa* L. ssp. *Japonica* cv. Nipponbare, WT) and *OsPIN2*-overexpressing transgenic plants (OX1, expression vector p1390-Ubi) (Chen *et al.*, 2012) were used in this study. Seeds were surface sterilized for 30min in a 10% (v/v) H_2_O_2_ solution, washed with deionized water, soaked in deionized water at 30 °C overnight, and germinated at 30 °C in darkness for 2 d. The germinated seeds were transferred to a net floating on a 0.5mM CaCl_2_ solution (pH 4.5) for 3 d. These seedlings were exposed to a 0.5mM CaCl_2_ solution (pH 4.5) containing 50 μM AlCl_3_ for 3 or 6h.

### Recovery treatments

The following recovery treatments were carried out: (i) Al treatment: seedlings were exposed to a 0.5mM CaCl_2_ solution (pH 4.5) containing 50 μM AlCl_3_ for 3h; and (ii) Al-Ca treatment: seedlings were exposed to a 0.5mM CaCl_2_ solution (pH 4.5) containing 50 μM AlCl_3_ for 3h and then transferred to 0.5mM CaCl_2_ (pH 4.5) for 1h.

### Brefeldin A (BFA) treatments

The following BFA treatments were carried out: (i) seedlings were exposed to a 0.5mM CaCl_2_ solution (pH 4.5) containing 35 μM BFA for 2h; (ii) seedlings were exposed to a 0.5mM CaCl_2_ solution (pH 4.5) containing 35 μM BFA and 50 μM AlCl_3_ for 3h; (iii) seedlings were exposed to a 0.5mM CaCl_2_ solution (pH 4.5) containing 50 μM AlCl_3_ for 3h and then transferred to a 0.5mM CaCl_2_ solution (pH 4.5) containing 35 μM BFA for 2h; and (iv) Seedlings were exposed to a 0.5mM CaCl_2_ solution (pH 4.5) containing 50 μM AlCl_3_ and 35 μM BFA for 3h and then transferred to 0.5mM CaCl_2_ solution (pH 4.5) for 1h.

### Microstructure observation

Root tips (0.3–1mm) were excised and fixed with 4% (w/v) glutaraldehyde and 3% (w/v) paraformaldehyde. They were washed three times with 0.1M PBS (pH 7.2) and post-fixed with 1% (w/v) OsO_4_ at –4 °C for 4h. The segments were washed three times again with the same buffer before being dehydrated in an ethanol series and embedded in epoxy resin. Ultrathin sections (70nm) were cut with ultramicrotome (Leica) and stained with lead citrate for 15min, followed by staining with uranyl acetate for 15min. Sections were studied by transmission electron microscopy (Tecnai 12, FEI) and energy-dispersive X-ray spectroscopy (Oxford EDS INCA Energy 300).

### Confocal laser scanning microscopic observation

For morin-only staining, root tips (0–5mm) were excised, washed with deionized water, and embedded in 5% agar, and then transversely sectioned at 0.2, 0.4, 0.6, 0.8, and 1mm from the apex with a vibratome (DTK-1000, Japan). Sections (50 μm) were collected and stained with 0.01% morin for 15min. For FM 4–64 and morin co-staining, seedlings were first exposed to 5 μM FM 4–64 for 30min, followed by Al or other treatments. After treatments, root tips (0–5mm) were excised after washing three times with deionized water, and embedded in 5% agar at 4 °C, and then transversely sectioned with a vibratome. Sections (50 μm) were collected and stained in 0.01% morin on ice for 15min and then washed with deionized water. The images were obtained immediately using confocal laser scanning microscopy (Carl Zeiss LSCM 780, Germany) at 488nm (morin) or 514nm (FM 4–64) excitation wavelength.

### Fractionation and determination of Al content

Fractionation and determination of the Al content in root tips were done as described by Huang *et al.* (2012). After treatments, root tips (0–8mm) or basal roots (8–16mm) were excised after washing three times with deionized water. For total Al determination, 80 root segments for each repeat were collected. For cell sap and cell wall Al determination, 160 root segments for each repeat were collected.

## Results

### Altered Al distribution in an *OsPIN2*-overexpressing line

**Fig. 1.**
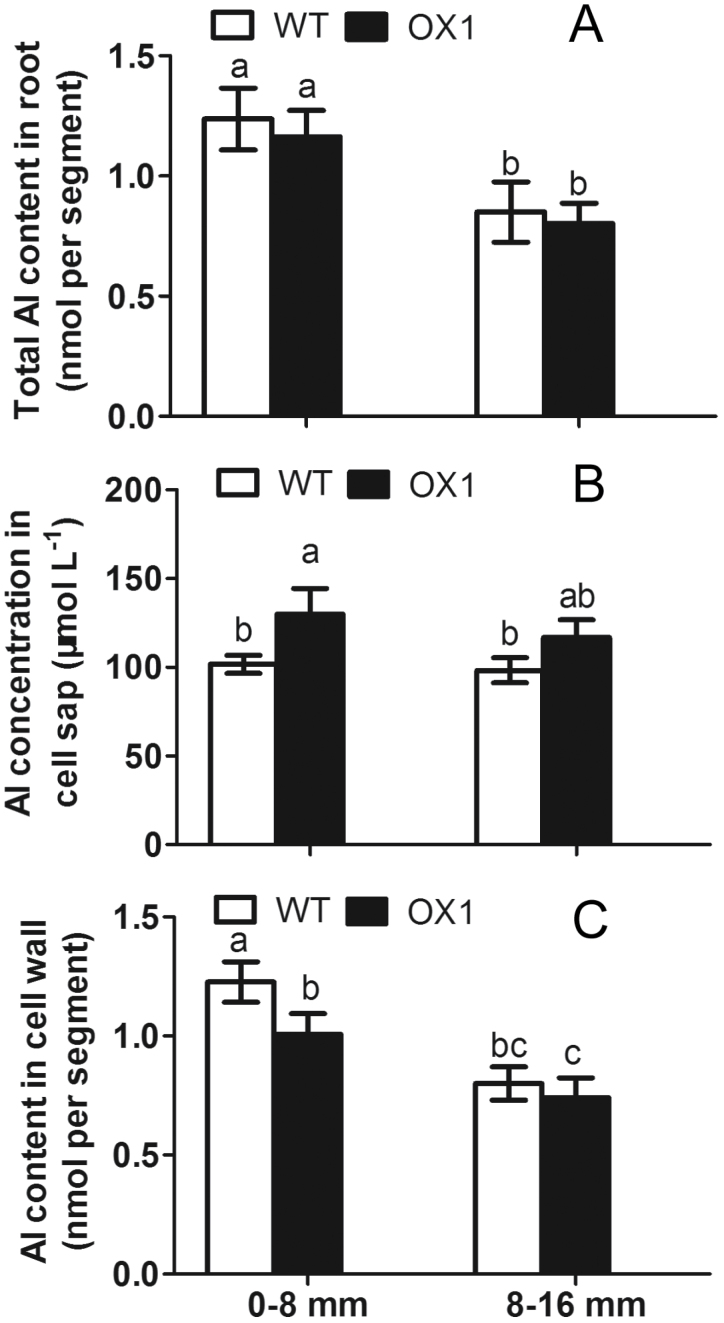
Al accumulation in WT and OX1 root apexes. Five-day-old seedlings of WT and OX1 were exposed to 50 μM AlCl_3_ for 6h. Root tips (0–8mm) and basal roots (8–16mm) were excised for determination. (A) Total Al content. (B) Al in the cell wall. (C) Al in the cell sap. Data are means±SE (*n*=3). Means with different letters are significantly different (*P*<0.05 by Tukey’s test).

Previous results showed that overexpressing *OsPIN2* could significantly increase Al internalization in the root apexes of rice relative to the WT (Wu *et al.*, 2014). Here, the differences in Al distribution in root tips (0–8mm) and basal roots (8–10mm) were further examined between OX1 and WT. The data showed that no significant differences in total Al content in either the root tips or the basal roots were observed in WT and OX1 after 6h exposure to Al (Fig. 1A). Compared with WT, OX1 only showed significantly increased Al concentration in the cell sap and reduced Al content in the cell wall in root tips but not in basal roots (Fig. 1B, C).

**Fig. 2.**
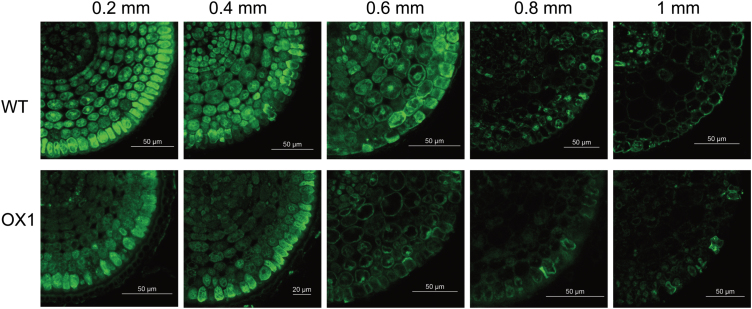
Cellular distribution of Al stained with morin (green). Five-day-old seedlings of WT and OX1 were exposed to 50 μM AlCl_3_ for 6h. Roots were transversely sectioned at 0.2, 0.4, 0.6, 0.8, and 1mm from the root apex for the morin staining and observation. (This figure is available in colour at *JXB* online.)

Morin forms a highly fluorescent green complex with Al and thus is widely used to sensitively localize Al in plant cells (Eticha *et al.*, 2005). To address the cellular Al distribution, morin staining was performed on the root apex sections in different regions. Al–morin fluorescence was clearly observed in these sections after 6h of Al exposure (Fig. 2). The fluorescence intensity inside the different root sections was: 0.2 mm>0.4 mm>0.6 mm>0.8 mm>1mm (Fig. 2). Notably, among different sections, morin staining showed a significant difference between WT and OX1. Further observation revealed that morin fluorescence was uniformly distributed in the different layers of cells in 0.2 or 0.4mm section of WT, whereas it occurred primarily in epidermal cells in these sections of OX1.

Eriochrome Cyanine R staining demonstrated that this Al-dependent staining occurred mainly in the root tip (0–1mm), and indeed was observed primarily in the epidermis and exodermis (Supplementary Fig. S1, available at *JXB* online). Similar to morin staining, the strongest Eriochrome Cyanine R staining was also observed in the 0.2mm section. Moreover, OX1 displayed stronger Eriochrome Cyanine R staining in root sections than WT at the same distance from the root apex. These results strongly suggested that overexpressing *OsPIN2* alters Al distribution in the root apex following short time exposures to Al.

### Root apex endocytosis might be involved in Al internalization

**Fig. 3.**
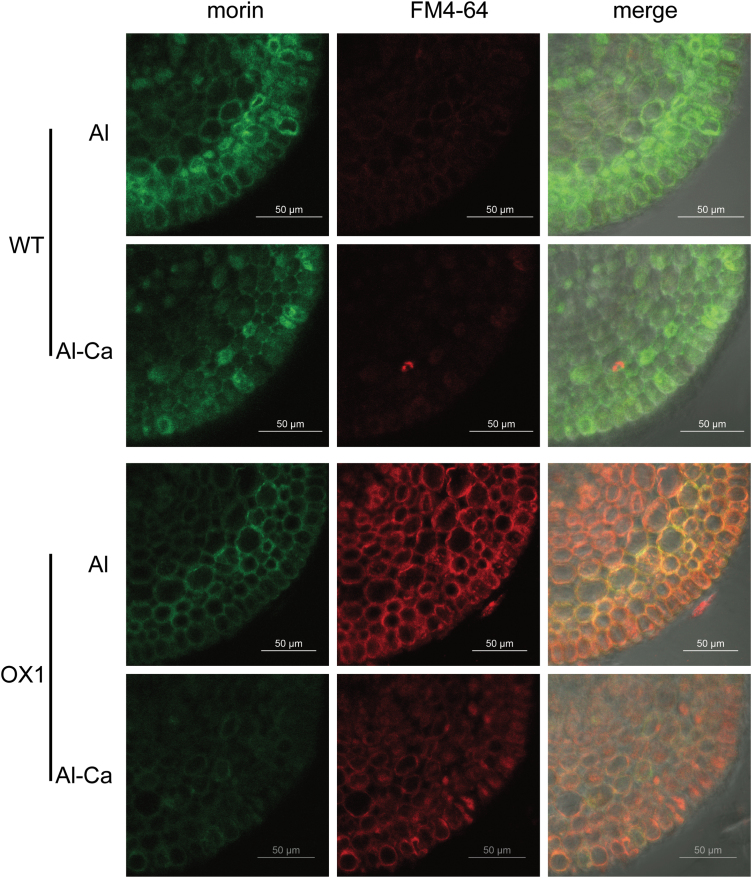
Changes in Al distribution (green) and vesicle trafficking (red) in recovery treatments. Five-day-old seedlings of WT or OX1 were pre-stained with 5 μM FM 4–64 for 30min and then transferred to Al or Al-Ca treatments (see Materials and methods). Roots were transversely sectioned at 0.5mm from the apexes for morin staining and observation. (This figure is available in colour at *JXB* online.)

FM 4–64, or *N*-(3-triethylammoniumpropyl)-4-{6-[4-(diethylamino) phenyl] hexatrienyl} pyridinium dibromide, belongs to a class of amphiphilic styryl dyes that are widely used for studying endocytosis and vesicle trafficking in living cells (Illéš *et al.*, 2006; Huang *et al.*, 2012). After 3h exposure to Al, OX1 displayed a stronger FM 4–64 fluorescence signal in the root apex cells than WT (Fig. 3), indicating that there was more frequent vesicle trafficking in OX1 apexes in comparison with WT. Notably, Al–morin fluorescence appeared to overlap with the FM 4-64-labelled compartment in the root cells of OX1. Moreover, this morin/FM 4–64 overlapping fluorescence became significant when OX1 seedlings were exposed to Al for 6h (Supplementary Fig. S2, available at *JXB* online), suggesting that vesicle trafficking might be involved in Al distribution in the cells of OX1 root apexes.

### Altered Al distribution in recovery treatment

**Fig. 4.**
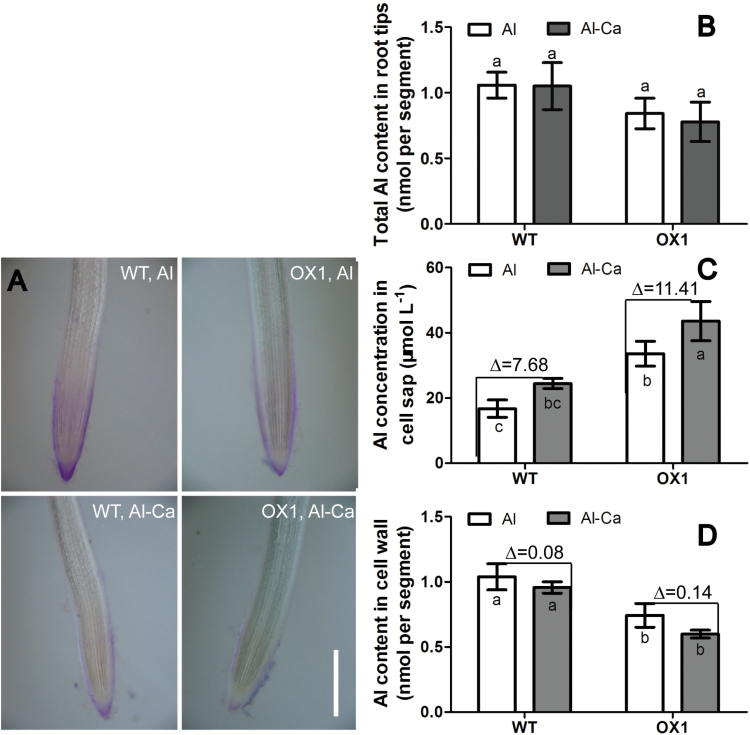
Changes in Al accumulation in recovery treatments. Five-day-old seedlings of WT or OX1 were transferred to Al or Al-Ca treatments (see Materials and methods). Root tips (0–10mm) were excised for determination. (A) Eriochrome Cyanine R staining. Bar, 500 μm. (B) Total Al content. (C) Cell wall Al content. (D) Cell sap Al content. Data are means±SE (*n*=3). The symbol ‘∆’ in C and D indicates the difference between Al treatment and Al-Ca treatment. Means with different letters are significantly different (*P*<0.05, Tukey’s test). (This figure is available in colour at *JXB* online.)

To investigate how Al distribution changes and what differences occur between OX1 and WT after the external Al stress is removed, a recovery treatment was also performed. After recovery treatment (Al-Ca), the morin signal and Eriochrome Cyanine R staining became weak in the root apexes of WT and OX1 (Figs 3 and 4A). However, the morin signal still could be observed clearly in the root cells of WT but was not detectable in those of OX1. These results indicated that Al distribution could be changed following a recovery treatment.

Further examination demonstrated that the changes occurred mainly in the Al content of cell walls (Fig. 4D) and the concentration in cell sap (Fig. 4C) but not in the total Al content in root apexes (Fig. 4B). When the Al was washed out, the Al content of the cell wall was decreased, and the reduction in OX1 apexes [∆=(OX1, Al) – (OX1, Al-Ca] was higher than that in WT root apexes [∆=(WT, Al) – (WT, Al-Ca)] (Fig. 4D). Instead, the Al concentration in cell sap was still increased although the external Al stress was removed (Fig. 4C). Moreover, a higher increase was found in OX1 apexes [∆=(OX1, Al-Ca) – (OX1, Al)] than that in WT root apexes [∆=(WT, Al-Ca) – (WT, Al)] (Fig. 4D).

### Impact of BFA on endocytosis and cellular Al distribution

**Fig. 5.**
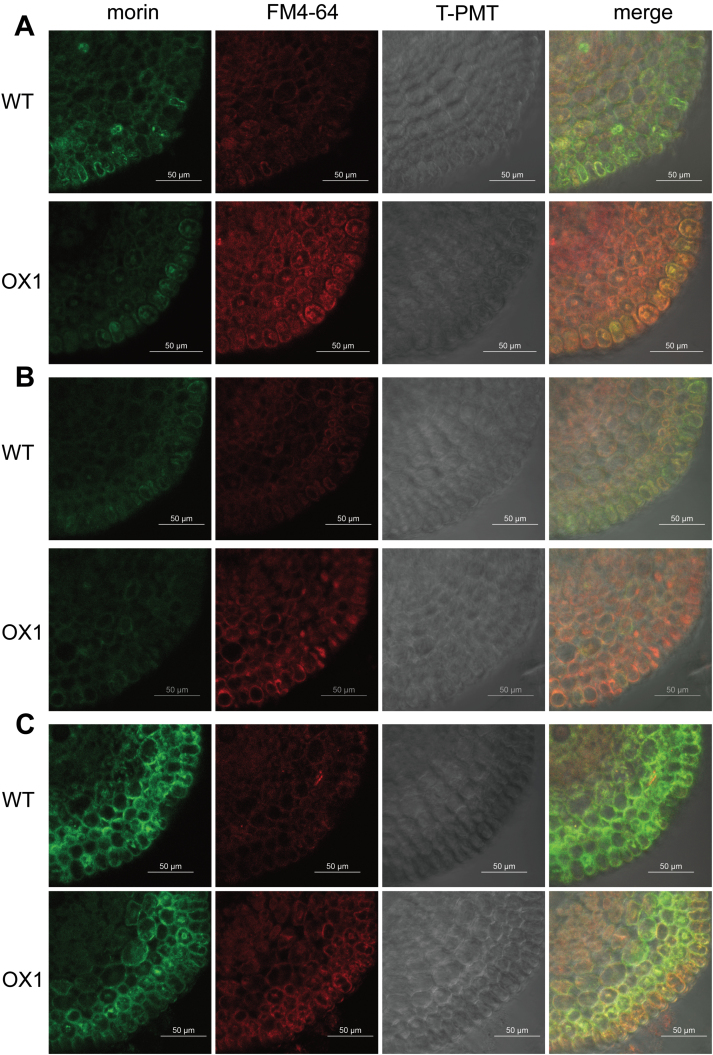
Effects of BFA on Al distribution (green) and vesicle trafficking (red). Five-day-old seedlings of WT and OX1 were pre-stained with 5 μM FM 4–64 for 30min and then exposed to 35 μM BFA+50 μM AlCl_3_ for 3h (A), 35 μM BFA+50 μM AlCl_3_ for 3h followed by 0.5mM CaCl_2_ for 1h (B), or 50 μM AlCl_3_ for 3h followed by 35 μM BFA for 2h (C). Roots were transversely sectioned at 0.5mm from the apexes for morin staining and observation. (This figure is available in colour at *JXB* online.)

BFA is an inhibitor of the plasma membrane recycling pathway and has also often been used as an inhibitor of vesicle trafficking (Nebenführ *et al.*, 2002). Since vesicle trafficking might affect Al distribution (Fig. 3 and Supplementary Fig. S2), the influence of BFA on vesicle trafficking and Al distribution in root apex cells was analysed. After 35 μM BFA treatment for 2h, FM 4–64 fluorescence was enhanced in the root apex cells of both WT and OX1; moreover, some regions, which were called BFA compartments, were observed more in cells of OX1 than in those of WT (Supplementary Fig. S3, available at *JXB* online). After exposure to the BFA and Al mixture for 3h, the root apex cells of both WT and OX1 displayed weak Al-dependent green fluorescent signal and a few areas of morin/FM 4–64 overlapping fluorescence occurred (Fig. 5A). After washing for 1h, BFA-induced compartments disappeared significantly in cells of OX1, and morin fluorescence became weaker (Fig. 5B). The Al-dependent green fluorescence, however, was still strong in the root apex cells of both WT and OX1 when they were exposed to Al for 3h and followed by BFA for 2h (Fig. 5C). It was found that weaker morin fluorescence, stronger FM 4–64 fluorescence, and more morin/FM 4–64 overlapping fluorescence occurred in OX1 root apex cells than in those of WT roots (Fig. 5C). These results demonstrated that BFA-induced inhibition of vesicle trafficking affects intracellular Al distribution.

### Multilamellar structures may contribute to Al internalization

**Fig. 6.**
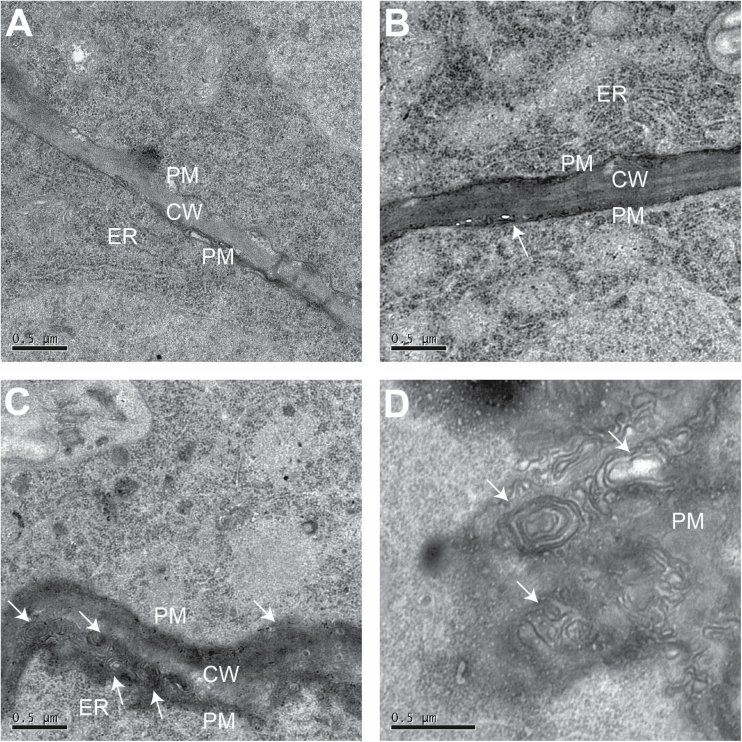
Effects of Al on the plasma membrane of epidermis cells. Five-day-old seedlings of WT and OX1 were exposed to 0 or 50 μM AlCl_3_ for 6h. Roots were transversely sectioned at 0.5mm from the apexes. (A) Section of WT without Al treatment. (B) Section of WT with Al treatment. (C, D) Sections of OX1 with Al treatment. Arrows in (C) and (D) show the multilamellar structures. CW, cell wall. PM, plasma membrane. ER, endoplasmic reticulum.

The differences in subcellular structure between the root apexes of WT and OX1 were further observed by transmission electron microscopy. Intriguingly, more multilamellar structures occurred near the cell wall or in the plasma membrane of epidermal cells in OX1 than in WT in the presence of Al (Fig. 6B, C). Moreover, parts of these multilamellar structures seemed to be isolated from the plasma membrane into the cell interior (Fig. 6D).

**Fig. 7.**
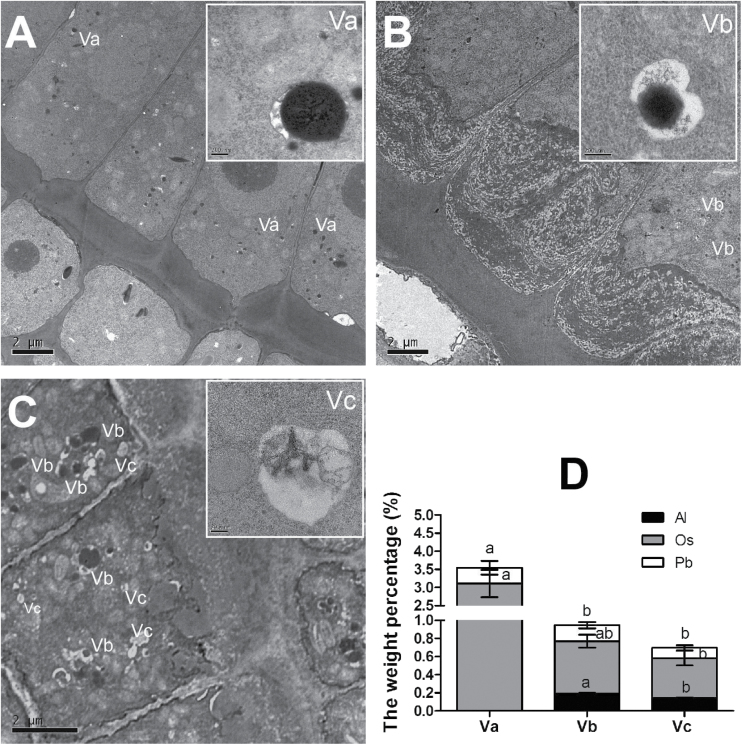
Effects of Al on vesicles in epidermis cells. Five-day-old seedlings of WT and OX1 were exposed to 0 or 50 μM AlCl_3_ for 6h. Roots were transversely sectioned at 0.5mm from the apexes. (A) Section of WT without Al treatment. (B) Section of WT with Al treatment. (C) Section of OX1 with Al treatment. (D) Weight percentages of Al, osmium (Os), and lead (Pb) in different vesicles. Va, vesicle A; Vb, vesicle B; Vc, vesicle C. Data are means±SE (*n*=3). Different letters in the same column indicate a significant difference (*P*<0.05, Tukey’s test).

A number of vesicles were also observed in the epidermal cells of OX1 or in those of WT with or without Al stress (Fig. 7). Further observations and energy-dispersive X-ray spectroscopy analysis revealed that they were different kinds of vesicles (Supplementary Fig. S4, available at *JXB* online). These were named vesicle A (Va), vesicle B (Vb), and vesicle C (Vc) on the basis of the differences in the structure and the content of lead (Pb), osmium (Os) and Al. Pb and Os, which are used as OsO_4_ and lead citrate in transmission electron microscopy pre-treatment, can be adsorbed by vesicles and are then termed osmiophilic granules (Kwiatkowska, 1973). The results showed that Va did look like an osmiophilic granule and accumulated Pb and Os (Fig. 7A, D). Vb was a vesicle accumulating Al, Pb, and Os and enclosing a black spot (Fig. 7B, D). In contrast to Vb, Vc accumulated less Al, Pb, and Os and enclosed a smaller floccule (Fig. 7C, D). Va only occurred in the epidermal cells without Al stress (Fig. 7A). In the presence of Al, some Vb could be observed in the epidermal cells of WT root apex (Fig. 7B). It is noteworthy that numbers of Vb and Vc were clearly observed in the epidermal cells of the OX1 root apex with Al treatment; moreover, some Vb and Vc aggregated to become a larger vesicle (Fig. 7C). These observations revealed that multilamellar structures and vesicles might contribute to the internalization of Al.

## Discussion

Al entering the root cell is an indispensable step for plants to tolerate Al toxicity internally. Several routes involved in transporting Al across the plasma membrane have been proposed. For mammalian and yeast cells, Al is transported across cell membranes by endocytosis (Exley, 2014). In plants, besides endocytosis (Vázquez, 2002; Illéš *et al.*, 2006; Shen *et al.*, 2008; Krtková *et al.*, 2012), some transporters, including Nrat1 and HmPALT1 (plasma membrane-localized Al transporting aquaporin 1) are responsible for transporting Al^3+^ across the plasma membrane (Xia *et al.*, 2010; Negishi *et al.*, 2012). Wu *et al.* (2014) showed that Al enhanced the expression of *Nrat1* in both WT and overexpressing *OsPIN2*, while the latter accumulated more Al; however, this does not necessarily mean that *Nrat1* contributes less to Al uptake. *OsPIN2::GUS* is expressed in epidermal cells in the meristem and transition zone (Supplementary Fig. S5B, C, available at *JXB* online) (Wang *et al.*, 2009). Both *OsPIN2* expression and its β-gluronidase (GUS) activity in the root tips were enhanced in response to Al (Supplementary Fig. S5). It was further found that overexpressing *OsPIN2* could significantly enhance Al internalization in the root tips but not in the basal roots (Fig. 1). These results were consistent with the possibility that *OsPIN2* is required for Al sensitivity in the rice apex.

Overexpression of *OsPIN2* resulted in less Al–morin fluorescence in root apex cells (Fig. 2) and less Eriochrome Cyanine R staining outside the epidermal cells (Supplementary Fig. S1). Moreover, using the Al marker morin and the endocytic marker FM 4–64, significant morin/FM 4–64 overlapping fluorescence was observed at 0.5mm from the root apexes of OX1, but not in those of WT (Fig. 3 and Supplementary Fig. S2). This important finding indicated that endocytosis might contribute to enhance Al internalization in root apexes of the overexpressing *OsPIN2* line (Illéš *et al.*, 2006; Amenós *et al.*, 2009; Krtková *et al.*, 2012).

The recycling of PINs between the plasma membrane and endosomes is controlled by subcellular vesicle trafficking (Kleine-Vehn *et al.*, 2008; Adamowski and Friml, 2015). They start their journey from the plasma membrane via the clathrin-mediated endocytic pathway, and reach the early endosomes or *trans*-Golgi network (TGN) (Lam *et al.*, 2007). From the early endosomes or TGN, two routes are involved in PIN subcellular transport. One is where PIN vesicles travel onwards to the pre-vacuolar compartment (PVC) or multivesicular body (MVB), and are finally degraded in a lytic vacuole (Nodzyński *et al.*, 2012). The other one is where PIN vesicles with TGN cargos or newly synthesized PIN proteins are first trafficked to the recycling endosomes, and then are secreted to the plasma membrane (Robinson *et al.*, 2008; Nodzyński *et al.*, 2012). BFA can inhibit the vesicle trafficking of PIN2 by interrupting the synthesis and/or secretion pathways of PIN2, which then leads to the formation of BFA-induced compartments (Nebenführ *et al.*, 2002; Kleine-Vehn and Friml, 2008; Jásik and Schmelzer, 2014). The results from BFA treatments indicated that BFA compartment formation might interfere with vesicle trafficking and then impede the internalization of Al (Fig. 5).

It was further found that Al accumulation in the root surface and cell wall was decreased after Al was removed from the solution (Fig. 4A, D), while Al concentration in cell sap was increased (Fig. 4C). Nevertheless, morin fluorescence became weak (Fig. 3). In contrast, the morin signal still could be observed clearly if BFA was applied after the removal of Al (Fig. 5C). Moreover, these changes were more sensitive in the root apexes of OX1 than in WT. These results support the results of Illéš *et al.* (2006), indicating that, although the outside Al toxicity was removed, cell-wall-bound Al still could be internalized into vacuoles and endosomes in the cells of the meristem and transition zone. Here, it was further demonstrated that overexpressing *OsPIN2* could positively regulate this process, while BFA might inhibit it.

Vázquez (2002) found that, after 96h exposure to 20 μM Al, a number of myelin structures, resembling multilamellar endosomes and containing Al–phytin, were observed between the cell wall and plasma membrane in the transition zone of the maize root apex (Baluška *et al.*, 2010; Baluška and Mancuso, 2013). In the current study, more multilamellar endosomes were also detected between the cell wall and plasma membrane in the root apex meristem of OX1 than in WT in the present of Al (Fig. 6). These multilamellar endosomes seemed to be isolated from the plasma membrane into the cytoplasm (Fig. 6D). Furthermore, after exposure to Al, some vesicles containing Al were identified in the root apex cells of both WT and OX1 (Fig. 7 and Supplementary Fig. S4). Strikingly, more vesicles with Al complexes occurred in the cells of OX1 than in WT. Moreover, in the cells of OX1, these vesicles aggregated to become larger vesicles, which closely resembled the PVC/MVB (Fig. 7C). These results may help us to understand why OX1 cells displayed a higher Al concentration in cell sap, whereas there was a weaker Al-dependent green fluorescent signal in the cytosol than for the WT in the root apex cells. These observations were also consistent with the possibility that vesicle trafficking is involved in Al internalization.

An important question arose from these results. How do vesicles carry out their role in Al internalization? It is generally accepted that pectin and hemicelluloses, two major components of the cell wall, are the primary binding sites for Al (Schmohl and Horst, 2000; Yang *et al.*, 2011; Zhu *et al.*, 2012). Pectin and xyloglucan (XyG), the major components of hemicellulose, are synthesized in the Golgi apparatus and are terminated to the wall surface via vesicles (Cosgrove, 2005). On the other hand, the internalization of cell wall pectin and XyG back into the meristematic and transition zone cells may also be important for proper assembly of the cell wall (Baluška *et al.*, 2002; Šamaj *et al.*, 2004; Baluška *et al.*, 2005; Mancuso *et al.*, 2007). Cell wall integrity is essential to maintain the polar distribution of PIN proteins at the plasma membrane (Adamowski and Friml, 2015). Moreover, internalized pectin and XyG seem to use the same vesicular trafficking as PIN1 and PIN2, or may act together with the PINs transporting auxin (Baluška *et al.*, 2002, 2005; Šamaj *et al.*, 2004; Mancuso *et al.*, 2007). Responding to Al stress, vesicle trafficking, which may function as a pectin and XyG transporter, is enhanced to modify the cell wall, which is bound by Al (Schmohl and Horst, 2000; Baluška *et al.*, 2002; Huang *et al.*, 2009). Also, Al treatment results in an increase in the content of pectin and hemicellulose in the root cell wall (Yang *et al.*, 2008). Moreover, in a previous study, it was found that not only was the content of pectin and hemicellulose increased but also the exudation of auxin was also increased in the roots of *OsPIN2* overexpression lines in the presence of Al (Wu *et al.*, 2014). In addition, a mutant of *XTH15* (xyloglucan endotransglucosylase/hydrolase 15), which make a contribution to cut or cut and rejoin XyG chains, demonstrates lower endogenous auxin levels and cell wall Al, but higher symplastic Al than the WT *Arabidopsis* (Zhu *et al.*, 2013). All these results support the hypothesis that there may be an intriguing relationship between cell wall remodelling, auxin transportation, and Al internalization. As multilamellar endosomes, which also occur in the pectin and XyG internalization pathway (Baluška *et al.*, 2005), could be observed in the apical meristem root cells of the *OsPIN2* overexpression line, it was hypothesize that Al may be internalized into the symplast by piggybacking onto the internalization of pectin and XyG (Panda *et al.*, 2009).

**Fig. 8.**
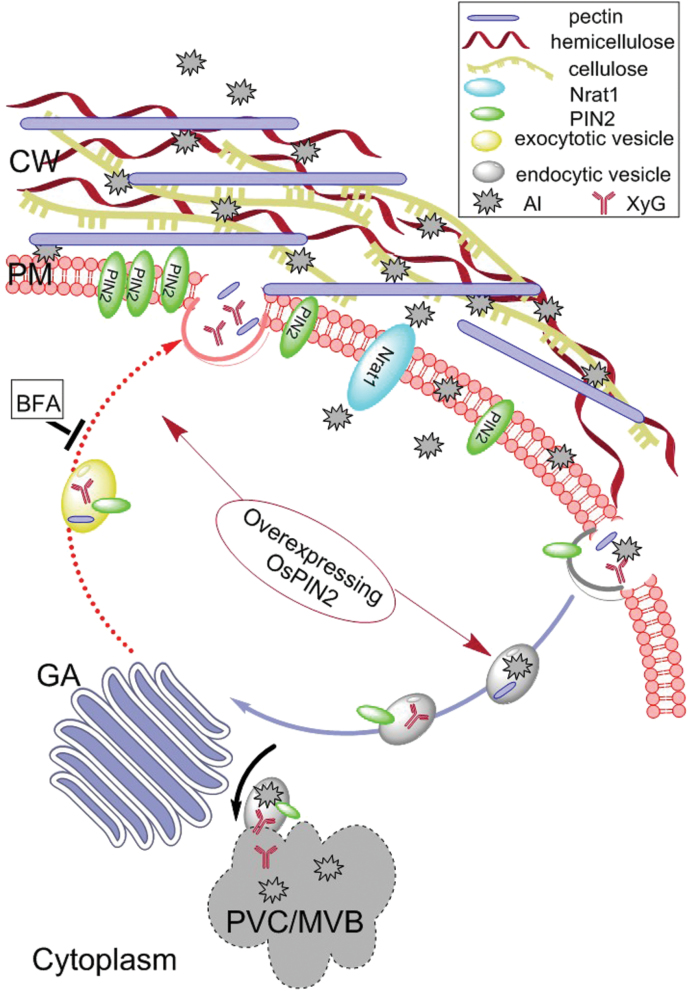
A plausible model, although requiring further validation, illustrates that overexpressing *OsPIN2* affects Al internalization by enhancing vesicular trafficking in the rice root apex. It is possible that overexpression of *OsPIN2* can enhance the frequency of vesicle trafficking, which contributes to the endocytosis and exocytosis of pectin and XyG. Subsequently, increased amounts of aluminium, which bind with pectin and XyG, are internalized via the endocytic trafficking of pectin and XyG. Furthermore, these endosomes tend to merge together and form the pre-vacuolar compartment (PVC). GA, Golgi apparatus. (This figure is available in colour at *JXB* online.)

Based on the results above, it was concluded that overexpressing *OsPIN2* enhanced Al internalization by elevating vesicular trafficking in the rice root apex. A plausible model was proposed as follows (Fig. 8). Al is firstly bound to pectin and XyG when rice roots suffer Al toxicity. To modify the cell wall, which is bound by Al, vesicle trafficking is enhanced to contribute to the endocytosis and exocytosis of pectin and XyG. In the pathway of endocytosis, the multilamellar endosomes with Al complexes, which may be a complex of PIN2, Al, pectin, or XyG, are first derived from the plasma membrane in the meristem of the OX1 root apex. These endosomes carrying Al are further internalized into the cytosol and, moreover, tend to merge together and form the PVC/MVB in which Al could not be detected by morin. In the pathway of exocytosis, overexpression of *OsPIN2* can also enhance the exocytotic trafficking of PIN2 which contributes towards stabilizing the abundance of PIN2 in the plasma membrane. By contrast, BFA can inhibit this exocytotic trafficking, and thereby attenuates the internalization of pectin and XyG because of the lack of PIN2 in the plasma membrane. Unfortunately, no method is currently available for identifying whether the Al complex is a complex of PIN2, Al, pectin, or XyG. Moreover, the possible mechanism of endocytic trafficking of PIN2 protein involving the internalization of pectin and XyG requires more investigations in the future work.

## Supplementary data

Supplementary data are available at *JXB* online.

Supplementary Fig. S1. Eriochrome cyanine R staining in different sections of root apex.

Supplementary Fig. S2. Al distribution and endocytosis co-stained with morin and FM 4–64.

Supplementary Fig. S3. The effect of BFA on endocytosis.

Supplementary Fig. S4. Energy-dispersive X-ray spectra acquired from the different kinds of vesicle in epidermal cell.

Supplementary Fig. S5. Effects of Al on *OsPIN2* and *OsPIN2::GUS* expression.

Supplementary Data

## Abbreviations:

Al: aluminium
BFA: brefeldin A
GUS: β-gluronidase
MVB: multivesicular body
OX: overexpression line
PVC: the pre-vacuolar compartment
TGN: *trans*-Golgi network
WT: wild type
XyG: xyloglucan.
