# Supplementary material for: Overexpressing OsPIN2 enhances aluminium internalization by elevating vesicular trafficking in rice root apex
Source: J Exp Bot. 2015 Aug 6;66(21):6791–801. doi: 10.1093/jxb/erv385 (PMC4623688; doi:10.1093/jxb/erv385)
Supplement: Supplementary Data [file supp_66_21_6791__index.html]

Overexpressing OsPIN2 enhances aluminium internalization by elevating vesicular trafficking in rice root apex — Overexpressing OsPIN2 enhances aluminium internalization by elevating vesicular trafficking in rice root apex — Supplementary Data 

# Overexpressing *OsPIN2* enhances aluminium internalization by elevating vesicular trafficking in rice root apex

## Supplementary Data

Data files

- Supplementary Data - Supplementary Data
