# Supplementary material for: Overexpressing OsPIN2 enhances aluminium internalization by elevating vesicular trafficking in rice root apex
Source: J Exp Bot. 2015 Aug 6;66(21):6791–801. doi: 10.1093/jxb/erv385 (PMC4623688; doi:10.1093/jxb/erv385)
Supplement: Supplementary Data [file supp_erv385_jexbot152686_file001.pdf]

# Supporting Information

## Extended materials and methods

### Real-time quantitative PCR analysis

5-d-old seedlings of WT or seedlings carrying *OsPIN2::GUS* were exposed to 0 or 50 μmol $L^{-1}AlCl_3$ for 6 h. For real-time quantitative RT-PCR analysis of *OsPIN2* gene expression, root apices (0-10 mm, 30 root each) were collected for RNA extraction using E.Z.N.A.® Plant RNA Kit (Omega, USA.). The first-strand cDNA was synthesized with PrimeScript® RT Master Mix (TaKaRa, Japan). The quantitative PCR was performed using Corbett Rotor-Gene 3000 Real-time PCR sytem with SYBR® Green PCR Master Mix (Life technologies, USA.). The primer sequence for RT-PCR of *OsPIN2* were F: 5-CAACACCTACTCCAGCCTC-3 and R: 5-TGGACCAGTCAAGAACCTC-3. *Histone H3* (F: 5-AGTTTGGTCGCTCTCGATTTC G-3 and R: 5-TCAACAAGTTGACCACGTCACG-3) was used as internal reference gene.

### GUS staining

5-d-old seedlings of *OsPIN2::GUS* transgenic plants (promoter-GUS cassette pBI101-P) (Wang *et al*., 2009) were exposed to 0 or 50 μmol $L^{-1}AlCl_3$ for 6 h. For root tip staining, root tips (0-10 mm) were excised for GUS staining. For root tip section staining, root tips (0-5 mm) were excised, washed with deionized water and embedded in 5% agar, then were transversely sectioned at 0.3 mm from apexes with vibratome (DTK-1000, Japan). 50 μm sections were collected, and stained with 0.1% Eriochrome Cyanine R. The images were obtained by light microscope.

### Eriochrome Cyanine R staining

Al accumulation in root surface was monitored using Eriochrome Cyanine R (Ma *et al*., 2004). For root tip staining, root tips (0-10 mm) were excised and stained with 0.1% Eriochrome Cyanine R. For root tip section staining, root tips (0-5 mm) were excised, washed with deionized water and embedded in 5% agar, then were transversely sectioned at 0.1, 0.2, 0.3, 0.4, 0.5, and 0.6 mm from apexes with vibratome (DTK-1000, Japan). 50 μm sections were collected, and stained with 0.1% Eriochrome Cyanine R. The images were obtained by light microscope.

## Supplemental Data

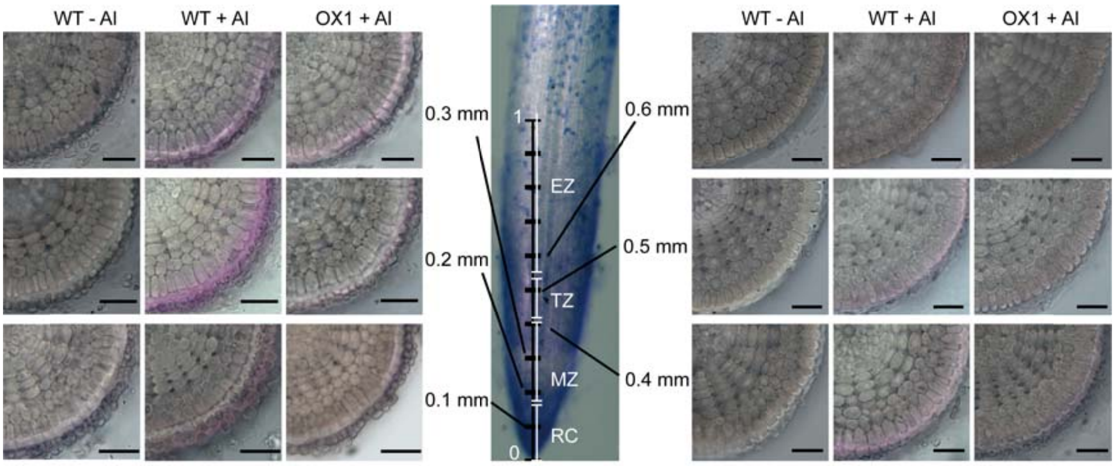


**Fig. S1.** Eriochrome cyanine R staining in different sections of root apex. 5-d-old seedlings of rice (WT and OX1) were exposed to 0.5 mmol $L^{-1}$ $CaCl_2$ (pH4.5) solution containing 0 or 50 μmol $L^{-1}$ $AlCl_3$ for 6 h. RC, root cap. MZ, meristem zone. TZ, transition zone. EZ, elongation zone. Bar = 50 μm.

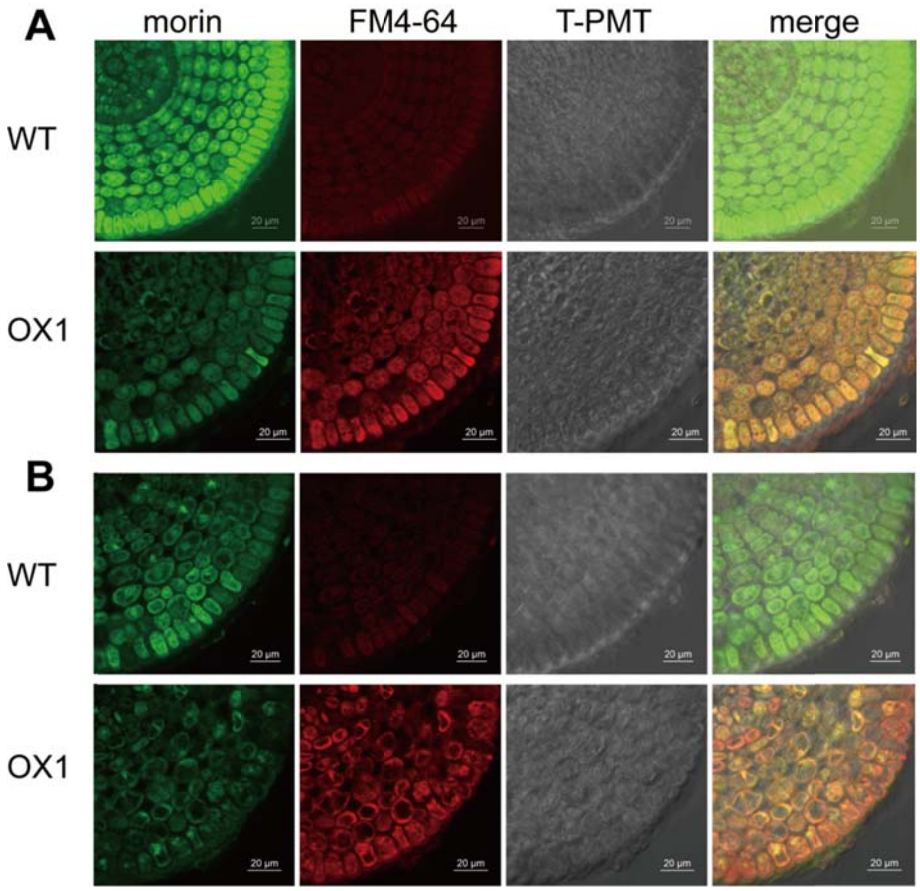


**Fig. S2.** Al distribution and vesicle trafficking co-stained with morin (green) and FM4-64 (red). 5-d-old seedlings of WT and OX1 were pre-stained with 5 μmol $L^{-1}$ FM4-64 for 30 min, and then exposed to 50 μmol $L^{-1}$ $AlCl_3$ for 6 h. Roots were transversely sectioned at 0.5 mm from apexes for the morin and FM4-64 co-staining.

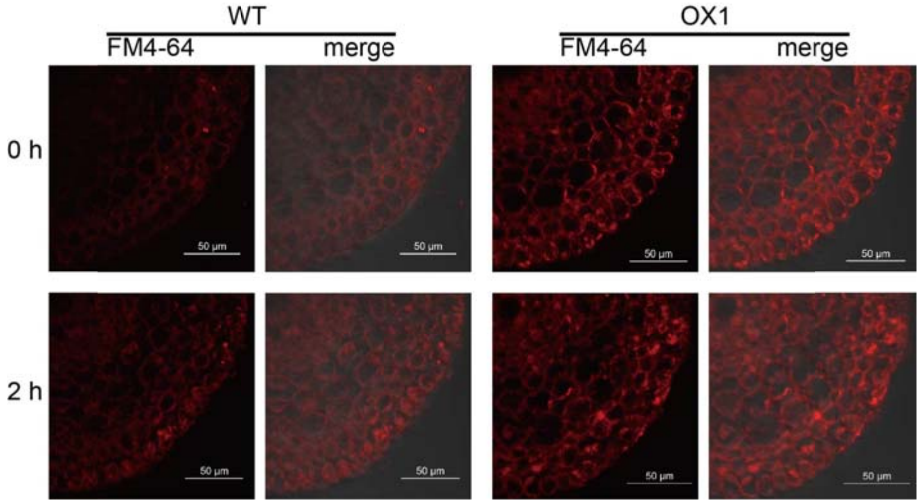


**Fig. S3.** The effect of BFA on vesicle traffickings (red). 5-d-old seedlings of WT and OX1 were pre-stained with 5 μmol $L^{-1}$ FM4-64 for 30 min, and then exposed to 35 μmol $L^{-1}$ BFA for 0 or 2 h. Roots were transversely sectioned at 0.5 mm from apexes for the FM4-64 staining.

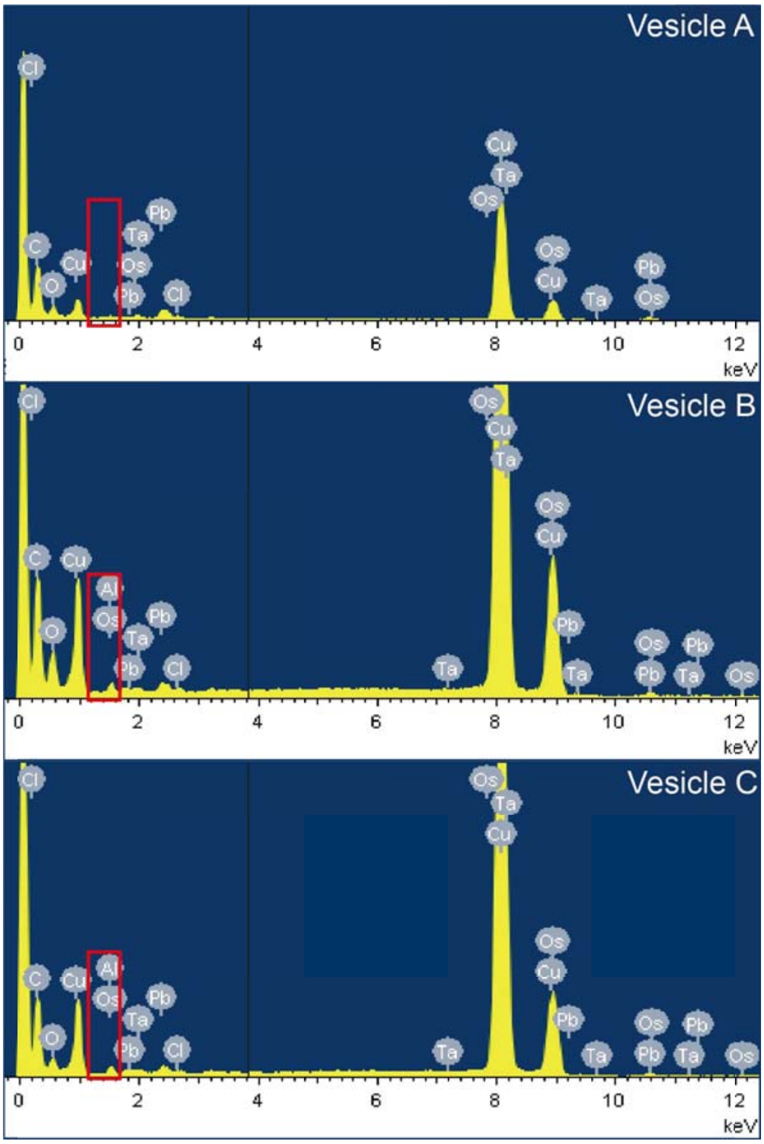


**Fig. S4.** Energy-dispersive X-ray spectra acquired from the different kinds of vesicle in epidermis cell. Red box shows the spectrum peak of Al. Pb and Os can be observed because they are used as $OsO_4$ and lead citrate in TEM pretreatment. Cu and Ta can be observed because they are the main components of Special Metal TEM Grids.

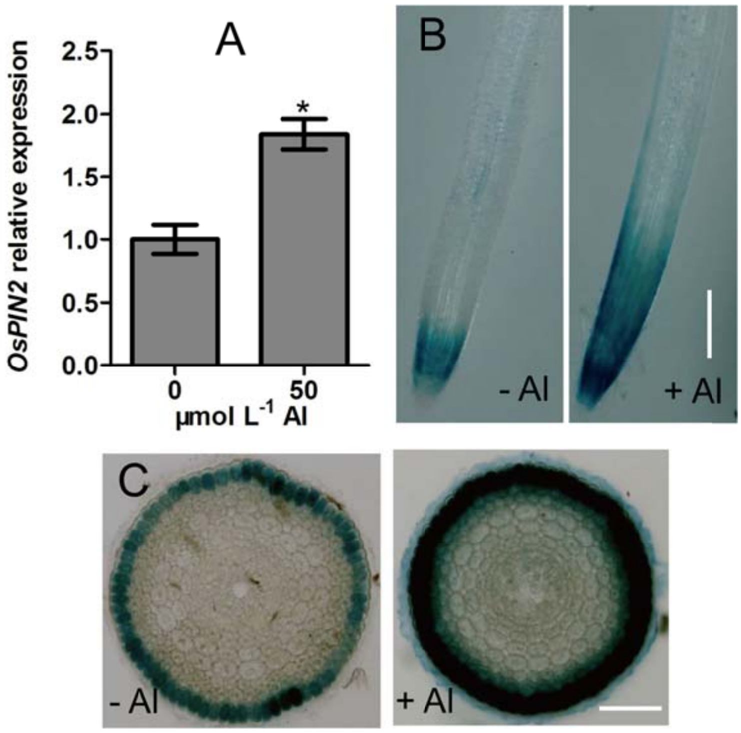


**Fig. S5.** Effects of Al on *OsPIN2* and *OsPIN2::GUS* expression. 5-d-old seedlings of WT or seedlings carrying *OsPIN2::GUS* were exposed to 0 or 50 μmol $L^{-1}$ $AlCl_3$ for 6 h. For real-time quantitative RT-PCR analysis of *OsPIN2* gene expression (A), root tips (0-10 mm) of WT seedlings were excised for RNA extraction. *Histone H3* was used as internal reference gene. Data are means ± SE (n=3). *is significantly different ($P < 0.05$, Student's *t*-test). For GUS staining, root tips (0-10 mm) of seedlings carrying *OsPIN2::GUS* were excised (B, bar = 500 μm), or roots were transversely sectioned at 0.5 mm from apexes (C, bar = 50 μm) for the GUS staining.
